# Assessment of V/Q mismatch during pressure support ventilation with electrical impedance tomography: a prospective physiological study

**DOI:** 10.1186/s40635-025-00837-6

**Published:** 2025-12-11

**Authors:** Mariachiara Ippolito, Giacomo Grasselli, Antonino Giarratano, Tommaso Mauri, Andrea Cortegiani

**Affiliations:** 1 Department of Precision Medicine in Medical, Surgical and Critical Care (Me.Pre.C.C), University of Palermo, 90127 Palermo, Italy; 2 Department of Anaesthesia, Intensive Care and Emergency, Policlinico Paolo Giaccone, Via del Vespro 129, 90127 Palermo, Italy; 3 Department of Pathophysiology and Transplantation, University of Milan, Via F. Sforza 35, 20122 Milan, Italy; 4 Department of Emergency, Foundation IRCCS Ca’ Granda Maggiore Policlinico Hospital, Milan, Italy

**Keywords:** Electrical impedance tomography, Acute respiratory distress syndrome, Assisted ventilation, V/Q matching

## Abstract

**Introduction:**

Spontaneous breathing may have both protective and negative effects in patients with ARDS, according to the severity of lung injury. Scarce evidence is available for physicians to safely guide the transition from controlled to assisted ventilation of ARDS patients. We aimed at describing variations of V/Q matching, measured with electrical impedance tomography (EIT), in patients recovering from ARDS, ventilated with different levels of pressure support.

**Methods:**

We performed a single-centre prospective observational study (*Clinicaltrial.gov*: NCT05781802), including adult mechanically ventilated patients admitted to the ICU with a diagnosis of ARDS according to the Berlin definition. The period of interest for the study was the transitioning phase from controlled to pressure support ventilation (PSV), and two observations were conducted. Data collection occurred at high and low pressure support, with each patient serving as his own control. The two conditions were defined according to a P0.1 threshold of 2 cmH2O (i.e. P0.1 < 2 cmH2O was considered "High PS" and P0.1 > 2 cmH2O was considered "Low PS"). The primary outcome was V/Q matching at the two different conditions.

**Results:**

We included a total of 15 patients receiving pressure support ventilation, after a median of 3 days of protective controlled ventilation. The median age was 69 y.o., and P/F at ICU admission was 132 [125–150] mmHg. The ΔPsupport difference between the two observations was 10 [10–10] cmH2O; pCO2 was 41 [37–47] mmHg at high support and 45 [41–50] mmHg at low support (*P* < 0.05), while tidal volume decreased (10.4 [9.8–11.9] ml/kg high; 8 [7.1–9] ml/kg low, *P* < 0.01). V/Q matching did not significantly differ from high pressure support (56.1% [46.4–69]) to low-pressure support (61.7% [56.7–69.5], *P* = 0.847). Still, nine patients improved V/Q matching at lower support, and the improvement between the two study steps was correlated with a higher PEEP level (*ρ* = 0.539, *P* = 0.038).

**Conclusions:**

Reducing the level of pressure support determined a redistribution of ventilation that did not, on average, result in improved V/Q matching compared to higher support. Our data underline the need for personalized settings during the transition from controlled to assisted mechanical ventilation in patients recovering from ARDS.

**Supplementary Information:**

The online version contains supplementary material available at 10.1186/s40635-025-00837-6.

## Introduction

Spontaneous breathing during mechanical ventilation can have both protective and negative effects on patient outcomes, according to the severity of lung injury [1]. In patients with severe ARDS, avoiding excessive inspiratory breathing efforts has an established protective role [2]. However, spontaneous breathing promotes the distribution of tidal volume towards dependent lung zones, and low levels of support pressure may determine a more homogeneous ventilation in patients recovering from ARDS, compared to higher support levels [3, 4]. Moreover, spontaneous breathing may enhance lung perfusion by reducing intrathoracic pressure, which lowers central venous pressure and increases venous return [1]. Gas exchange improvement in experimental lung injury models during pressure support vs. controlled ventilation can be explained by the redistribution of lung perfusion to nondependent lung areas and improvement of V/Q matching even in the absence of significant lung recruitment [5, 6]. Clinical studies have shown that failed attempts to switch patients to assisted ventilation are associated with significantly worse clinical outcomes [7]. The effects of spontaneous breathing are linked to and mediated by patient effort. Indeed, during the early phases of assisted ventilation, the level of patient effort may vary significantly, potentially leading to pathophysiological changes, such as patient self-inflicted lung injury in cases of excessive effort, or impaired ventilation–perfusion matching and diaphragmatic atrophy in cases of prolonged insufficient effort [8, 9]. Electrical impedance tomography has been clinically used as a non-invasive tool to assess V/Q matching [10] in patients with ARDS [11, 12]. Although it is mainly validated in controlled mechanical ventilation, it has been proven as feasible during spontaneous breathing, provided that a breath holding of sufficient duration avoids any alteration to the measurements [10]. This study aims to describe differences in the regional distribution of ventilation and lung perfusion (and thus in V/Q matching) in patients recovering from ARDS, during pressure support ventilation at different effort levels and using electrical impedance tomography.

## Methods

We conducted a single-centre prospective observational study in a tertiary hospital in Palermo, Italy. The protocol was prospectively registered in Clinicaltrials.gov (NCT NCT05781802). Patients were screened from a 17-bed general ICU and included in the study in the period from February 2023 to November 2024. The study was approved by the local Ethics Committee (EC Palermo 1 n° 08/2022, 14 Sep 2022). Written informed consent was obtained from included patients or their legal representatives according to national regulations.

We included consecutive invasively ventilated adult patients admitted to the ICU with a diagnosis of ARDS according to the Berlin definition [13] and with a central line placed in the internal jugular vein. Exclusion criteria were the presence of any contraindication to EIT monitoring (Pulmovista 500 Draeger), cardiogenic pulmonary oedema, pulmonary embolism, COPD or asthma exacerbation, pre-existing diaphragmatic function impairment, neuro-muscular diseases or impairment, recommendation for limitation of care or expected survival < 48 h according to the treating physician. The primary outcome was variation in V/Q matching, measured as the percent variation of regions with coexisting ventilation and perfusion (defined as V/Q matching all through the manuscript) at two different ventilatory conditions (“high” level of pressure support and “low” level of pressure support).

### Data collection procedures

The two observation conditions in the study were:

- High PS: high pressure support
- Low PS: low pressure support

Each patient served as his own control.

The two conditions were defined according to a P0.1 threshold of 2 cmH2O (i.e. P0.1 < 2 cmH2O was considered "High PS" and P0.1 > 2 cmH2O was considered "Low PS").

Data collection occurred during the first day of transition from controlled to pressure support ventilation upon the decision of the treating physician not involved in the study protocol. The pressure support level was maintained for at least 20 min before data collection, and the sedation level was kept consistent between the two timepoints.

In detail, once the patients transitioned from controlled to pressure support ventilation, P0.1 was measured at the clinically selected PS level. The clinically selected PS level was classified as “High” if P0.1 was found < 2 cmH2O, as the median of three measurements, or “Low”, if P0.1 was found > 2 cmH2O. All the measurements were conducted under the clinical condition and then repeated after at least 20 min of ventilation under a different PS. This second condition was reached by increasing or decreasing PS in steps of 5cmH2O, until the value of P0.1 reached the opposite side of the threshold.

P0.1 was measured as the fall in Paw during the first 100 ms of an end-expiratory occluded pause, determined by a spontaneous inspiration.

The measures were conducted using Dräger Evita® V800, and the median of three measurements was adopted.

The threshold of P0.1 = 2 cmH₂O was selected based on the methodology of a previous study conducted in a similar patient population [4].

Data collection included ventilatory settings, plateau airway pressure (Pplat) measurement after a 3-s inspiratory pause, pressure muscle index (PMI) calculated as the difference between the Pplat and the total airway pressure, occlusion pressure (ΔPocc) measurement during an expiratory pause [14], data on gas exchange and EIT data recording for 180 s. Pplat was considered reliable when measured at the end of a 3-s inspiratory hold pause without any visually inspected muscular activity of the patients and at zero flow [15, 16]. During the EIT recording, a 10-s inspiratory pause was performed, and a bolus of NaCl 5% 10 ml was administered via central line in the internal jugular vein to allow offline analysis of ventilation/perfusion matching, following previously validated methods [10, 11]. Sedation was kept constant during the measurements, and, in cases of conscious patients, we explained the procedure and instructed the conscious patients to avoid movements or efforts during the hold. The entire procedure was conducted by at least two investigators to allow for a visual inspection of the muscular activity of the patients and the flow/time curve at the ventilator during the hold. At the end of the measurement, the morphology of the impedance/time curve was also inspected, and in case of alterations, technical errors or interruptions in the impedance curve were observed, the measures were repeated a maximum of once in the following 30 min. If issues persisted, the patients were excluded from the study.

At the end of all the measurements, clinically selected settings of the ventilator were restored.

We also collected data on patients’ vital parameters at each time point, including cardiac index (CI). In patients already monitored with a calibrated transpulmonary thermodilution method, cardiac index was recorded and recalibrated at each data collection time point. In patients without advanced haemodynamic monitoring, CI was estimated using the ultrasound-based LVOT VTI method.

We also collected data on patients’ characteristics at ICU admission and ICU and hospital mortality.

### Data analysis and sample size justification

Offline EIT data were extracted and analysed using Draeger EIT analysis tool v6.3 and an application for perfusion analysis provided by “Dipartimento di Fisiopatologia Medico-Chirurgica e dei Trapianti”, University of Milan [10, 11]. The application provided data into an electronic dataset per patient, and a graphical visualization of patient data (see Figure S1, Supplementary Material 1). Data were analysed using descriptive statistics and presented using median and interquartile range, as appropriate. Wilcoxon signed-rank test was used to compare outcome measures at the different timepoints, and Hodges–Lehmann estimator of the paired difference along with its 95% confidence interval was also reported. A post hoc analysis was conducted looking for correlations between the percent variation in V/Q matching and the level of PEEP, adopting the Spearman test, as appropriate. *P* = 0.05 was the assumed threshold for significance for all the analyses. Data were analysed with JASP (Version 0.18.3). Figures 2 and 3 were created using RStudio, version 2024.12.0 + 467, packages *ggplot* and *base*. As no data were available on EIT measurement of V/Q matching in patients with ARDS under pressure support invasive ventilation at the protocol drafting stage, the sample size for this physiological study was selected as a convenience sample size for explorative purposes. The enrolment of a total of 15 consecutive patients completing all the timepoints was considered reasonable to perform a descriptive analysis on V/Q matching.

## Results

A total of 25 patients were considered eligible during the study period. Of these, 5 were excluded due to technical issues (incorrect data saving, poor data quality) and 5 due to the concomitant presence of COPD, for a total of 15 patients included in the study (flow diagram showing the inclusion and exclusion process is shown at Figure S2, Supplementary Material 1). The patients had a median age of 69 years and a PaO_2_/FiO_2_ at ICU admission of 132 mmHg [125–150]. Twelve patients (80%) had a primary lung injury as the cause of ARDS, while the remaining 20% developed ARDS as secondary to an extrapulmonary condition. ICU mortality was 53%. Seven patients were discharged alive from ICU and hospital. Radiological findings and the cause (proven or most likely) of ARDS are listed in Supplementary Material 1, Table S1. Table 1 summarizes the patient characteristics at ICU admission. Table S2 (Supplementary Material 1) shows ventilation and perfusion data from EIT data analysis conducted at baseline under controlled ventilation.

**Table 1:** Patients’ characteristics at ICU admission

Age, y	69 [61–71.5]
RBW, kg	70 [60.5–74.25]
Height, cm	165 [160–170]
PaO_2_/FiO_2,_ mmHg	132 [125–150]

Data are reported as median [IQR]

The median duration of volume-controlled ventilation before switching to pressure support ventilation was 3 days [2–5.5]. In 5 cases, the clinically selected pressure support level resulted in a P0.1 > 2 cmH2O and was thus the first measurement condition, classified as “low” pressure support. In the remaining 10 cases, the clinically selected pressure support level was associated with a P0.1 < 2 cmH2O and was classified as “high” pressure support. Each patient subsequently underwent a second measurement at the *opposite* level of pressure support: the 5 initially in the “low” group were reassessed at “high” support, and the 10 initially in the “high” group were reassessed at “low” support.

Regardless of the order of measurement, patients ventilated with high pressure support (median 15 cmH₂O [14–19]) received a median tidal volume normalized to predicted body weight (Vt/PBW) of 10.4 ml/kg [9.8–11.9]. Under low pressure support (5 cmH₂O [4–10]), the Vt/PBW was significantly lower, at 8 ml/kg [7.1–9.0] (*P* < 0.001). When ventilated with low pressure support, patients showed a higher respiratory rate (*P* = 0.06) and higher PaCO₂ (*P* = 0.05), while both plateau pressure (Pplat) (*P* = 0.02) and driving pressure (*P* = 0.04) were significantly lower compared to ventilation with high pressure support. Spontaneous effort measures varied consensually with the level of support, as expected. There were no significant differences in haemodynamic parameters, sedation levels, or electrolytes. Data collected under the two ventilation conditions are summarized in Table 2.

**Table 2:** Measured variables and outcome at high and low pressure support

	High pressure support	Low pressure support	*P* value
Ventilation			
VT, ml	628 [536–750]	423 [389–578]	< 0.001*
VT/PBW, ml/kg	10.4 [9.8–11.9]	8.0 [7.1–9.0]	< 0.001*
Ppeak_aw_, cmH_2_O	27 [25–30]	17 [15–22]	< 0.001*
ΔPsupport, cmH_2_O	15 [14–19]	5 [4–10]	< 0.001*
PEEP, cmH_2_O	10 [8–12]	10 [8–12]	NA
VM, ml	8470 [7097–9557]	7826 [6848–9278]	0.229
RR, bpm	14 [11–16]	16 [14–24]	0.006*
Pplat, cmH_2_O	25 [21–27]	20 [19–24]	0.022*
Pdriving, cmH_2_O	15 [12–16]	12 [9–16]	0.041*
Pmean, cmH_2_O	14 [13–17]	12 [11–15]	< 0.001*
CRS, ml/cmH_2_O	47 [35–64]	42 [35–48]	0.055
P0.1, cmH2O	− 1.4 [− 1.7 to − 0.9]	− 3 [− 3.3 to − 2.9]	< 0.001*
PMI, cmH_2_O	− 2 [− 5 to − 0.5]	5 [4–6.5]	< 0.001*
DeltaPocc	− 8 [− 13.6 to − 4.5]	− 13 [− 16 to -12]	0.005*
Gas exchange and arterial blood gas			
SaPO_2_, %	98 [95–99]	97 [94–98]	0.078
PaO_2_, mmHg	110 [87–141.5]	103 [99–110]	0.187
FiO_2_, %	0.4 [0.4–0.5]	0.4 [0.4–0.5]	NA
Vent. ratio	1.53 [1.29–1.77]	1.57 [1.43–1.86]	0.706
P/F, mmHg	260 [203–307]	224 [206–284]	0.177
pH	7.45 [7.42–7.49]	7.42 [7.39–7.48]	0.035*
PaCO_2_, mmHg	41 [37–46.5]	45 [41–50.5]	0.005*
HCO_3_−, mmol/l	29 [26–31]	29 [26–33]	0.147
Lac, mmol/l	1.2 [0.8–1.4]	1.2 [0.85–1.35]	0.172
Hb, g/dl	9.2 [8.3–11.8]	9.2 [8.6–11.6]	0.944
Haemodynamics, fluids, sedation			
SBP, mmHg	127 [114–132]	123 [111.5–130.5]	0.887
DBP, mmHg	59 [52–63]	59 [50–62.5]	0.462
MAP, mmHg	82 [73–87]	77 [73–87]	0.711
HR, bpm	85 [65–92]	81 [69–87]	0.889
TEMP, °C	36 [36–37]	36 [36–37]	0.424
CVP, mmHg	11 [9–12]	9 [7–12]	0.057
C.I, L/min/m2	3.5 [2.2–4.1]	3.2 [2.0–4.0]	0.624
RASS	− 3 [− 3 to − 2]	− 3 [− 3 to − 2]	NA
BPS	3 [3–3]	3 [3–3]	NA
Na+, mmol/l	144 [137–145]	143 [138–146]	0.097
Cl−, mmol/l	109 [104–110]	108 [105–110]	0.305
Fluid balance, ml	− 143 [− 1065 to -340]	− 143 [− 1065 to -340]	NA
Primary outcome			
V/Q matched regions, %	56.1 [46.4–69.0]	61.7 [56.7–69.5]	0.847

Data are reported as median [IQR] and percentages

**Fig. 1:**
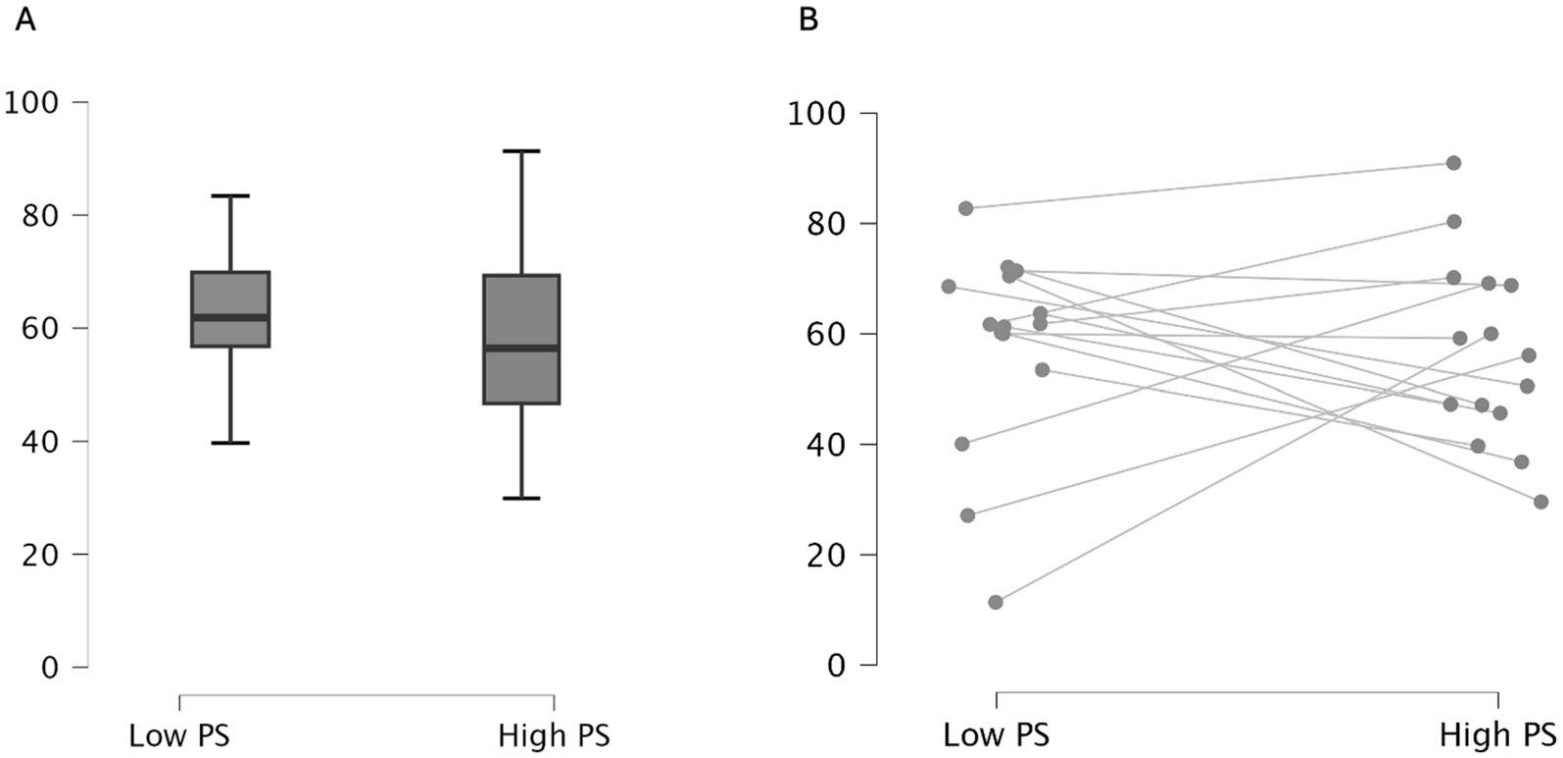
Boxplots (panel **A**) and spaghetti plot (panel **B**) showing V/Q matching under low pressure support and high pressure support

The V/Q matching was higher at low pressure support compared to high pressure support (61.69 [56.7–69.53] vs. 56.1 [46.4–69]) but without statistical significance (Hodges–Lehmann estimate: − 2.645; 95% confidence interval for Hodges–Lehmann estimate: lower− 16.251; upper 13.449; *P* = 0.847) (Table 2**, **Fig. 1). A post hoc sensitivity analysis (leaving in–leaving out) excluding the patients with outlier V/Q matching values from the dataset (see Supplementary Material 1), did not significantly affect the results, showing no significant differences between the two conditions (High pressure support: 50.5 (45.7–69.1); low pressure support: 61.8 (60.2–70.5); *P* = 0.244).

**Fig. 2:**
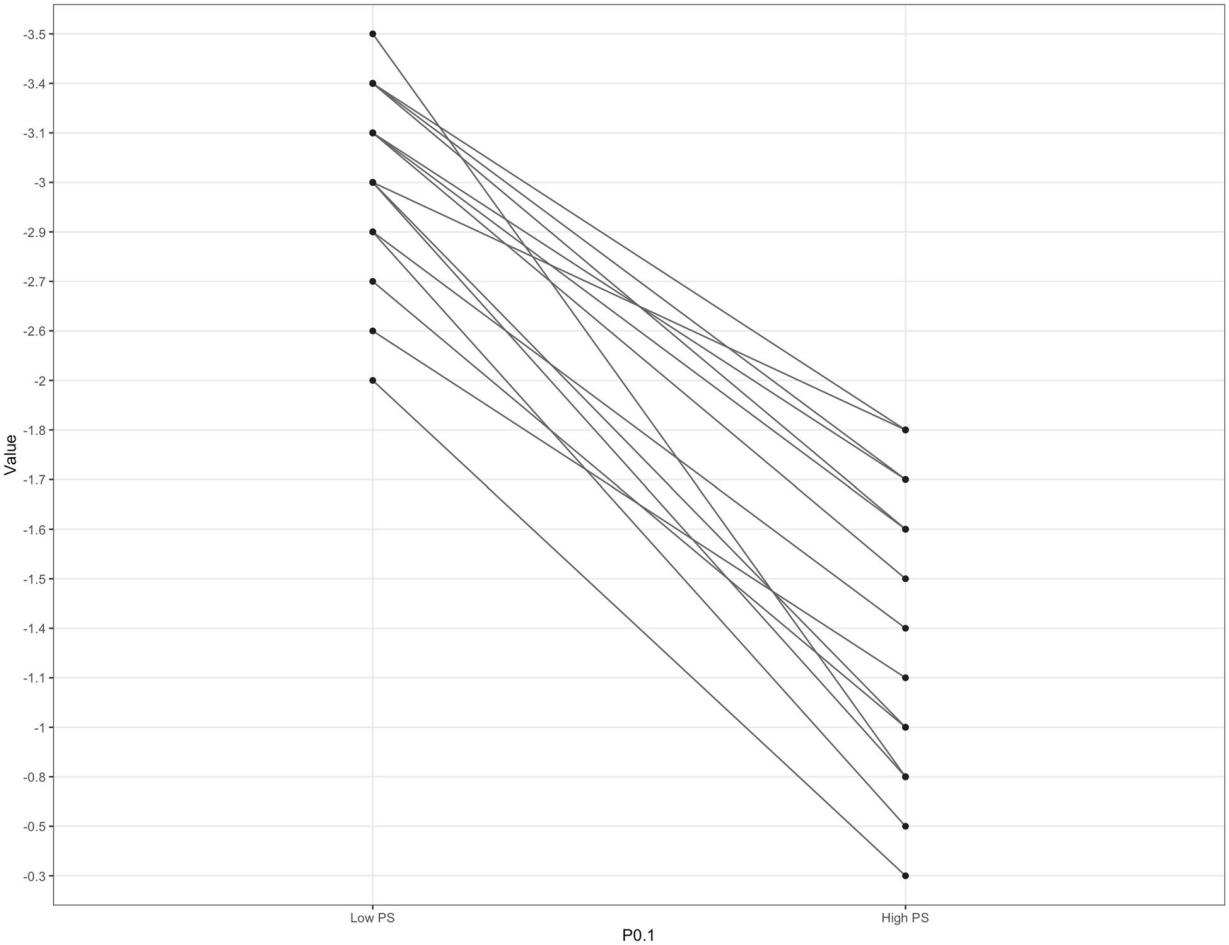
Plot representing the values of P0.1 reached by each patient at the two measurement conditions (i.e. low and high pressure support)

Figure 2 shows the values of P0.1 reached by each patient at the two conditions of measures.

Concerning the regional analysis of ventilation, low pressure support was associated with an increased percentage of ventilation distributed towards dorsal regions (47.7 [38.1–53.9] vs. 42.1 [38.1–48.1], *P* = 0.05) and a decreased percentage of ventilation distributed towards ventral regions (52.3 [46.1–61.9] vs. 57.9 [51.9–61.9], P = 0.05) (Table 3). Concerning the regional analysis of perfusion, low pressure support showed an increased percentage of perfusion distributed towards ventral regions (57.8 [41.7–68.7] vs. 54.4 [38.7–59.6], *P* = 0.489) and a decreased percentage distributed towards dorsal regions (42.2 [31.3–58.3] vs. 45.5 [40.4–61.2], *P* = 0.489), without reaching statistical significance. Distribution of ventilation and perfusion according to regional V/Q matching is reported in Supplementary Material 1, Table S3.

**Table 3:** Ventilation and perfusion distribution results from EIT data analysis

	High pressure support	1 st quartile	3rd quartile	Low pressure support	1 st quartile	3rd quartile	*P* value
V ventral (non dep) %	57.9	51.9	61.9	52.3	46.1	61.9	0.005*
Q ventral (non dep)%	54.4	38.7	59.6	57.8	41.7	68.7	0.489
V dorsal (dep) %	42.1	38.1	48.1	47.7	38.1	53.9	0.005*
Q dorsal (dep) %	45.5	40.4	61.2	42.2	31.3	58.3	0.489
Only V—dead space %	29.4	19.5	46.3	32.4	17.8	36.0	0.847
Only Q—shunt %	11.4	4.4	16.4	10.4	2.6	14.0	1.000

Data are reported as median and quartiles

**Fig. 3:**
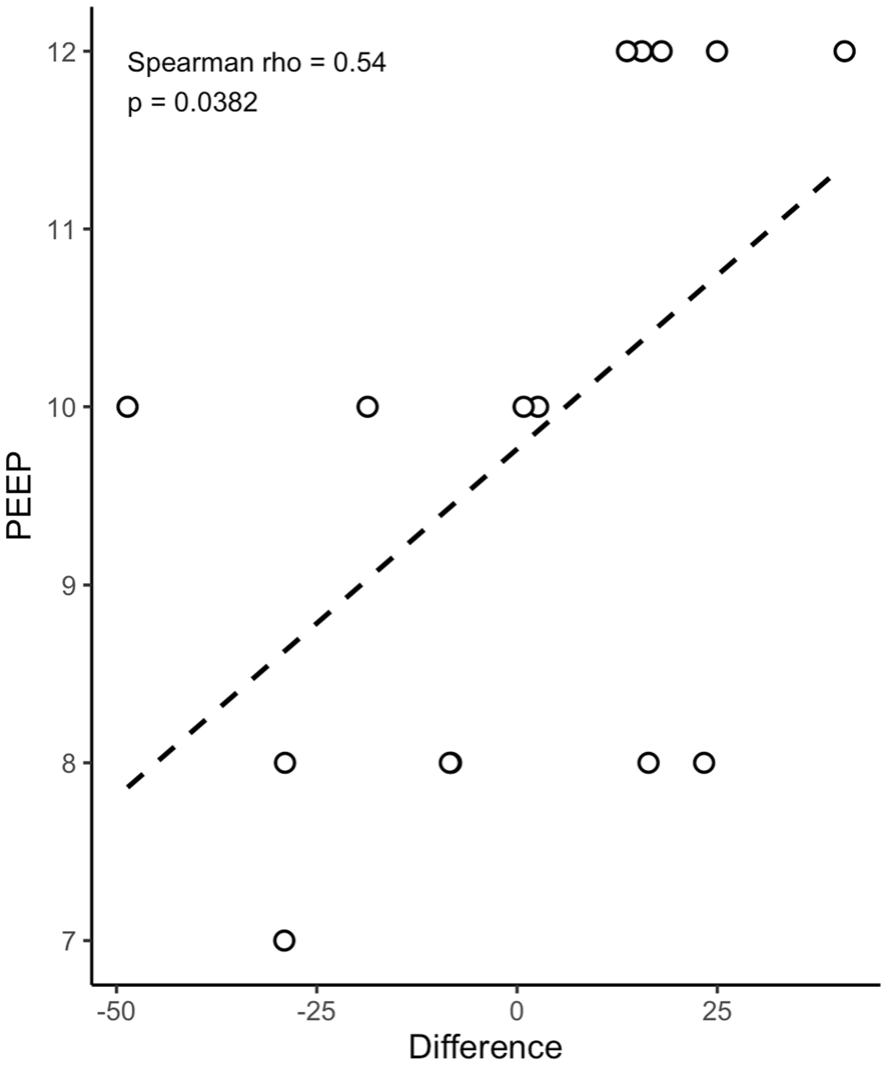
Scatter plot of distribution of V/Q matching difference (V/Q at low support minus V/Q at high) according to PEEP level

V/Q matching appeared to improve more consistently from high to low pressure support conditions at higher levels of PEEP (Spearman *ρ* = 0.539, *P* = 0.038, Fig. 3).

## Discussion

The main finding of this study is that reducing the level of pressure support determined a redistribution of ventilation that did not, on average, result in improved V/Q matching compared to higher support. We observed a change of ventilation distribution from ventral to dorsal regions during low pressure support, compared to high level of pressure support, but this did not lead to a significant overall change of V/Q matching between the two conditions. We also noted a modification of perfusion distribution towards the ventral region, as also suggested by preclinical data [6], but statistical significance was not reached for this observation.

Historical reports have already investigated changes in gas exchanges and V/Q matching at different conditions of mechanical ventilation. Putensen et al. conducted a trial randomly assigning patients to receive PSV and APRV with and without spontaneous breathing, maintaining equal minute ventilation or equal airway pressure limits. They found better V/Q matching during APRV with spontaneous breathing in patients with ARDS compared with APRV without spontaneous breathing, while PSV did not improve V/Q distributions when compared with controlled mechanical ventilation [17].

Mauri et al. conducted in 2012 a physiological crossover study on 12 patients with ARDS and showed that pressure support ventilation promoted dorsal ventilation compared to controlled ventilation, especially at lower levels of support [4]. However, they did not assess perfusion or V/Q matching directly. Preclinical studies showed a potential benefit of spontaneous breathing on V/Q matching in preclinical models [6].

Incorporating V/Q matching assessment as a complementary monitoring tool, alongside traditional parameters like measures of respiratory effort measures, tidal volume, and gas exchange, could provide clinicians a more comprehensive understanding of the pathophysiology of the disease during transition from controlled to assisted ventilation. Overall, a non-significant trend towards an improvement in V/Q matching was observed in our cohort and we cannot exclude it was observed by chance. Moreover, it remains uncertain whether an improved V/Q matching would be associated with better clinical outcomes.

We also performed a post hoc analysis to explore the pathophysiologic mechanisms underlying the variability in V/Q matching between the two pressure support levels in the patients included in our cohort. We found a significant correlation between the difference in V/Q matching and the PEEP level, which was kept constant within each patient but varied across individuals in the study. This observation may suggest that the effects of low pressure support on V/Q matching are influenced by the PEEP setting. However, this result spans a range of PEEP differences of 4 cmH_2_O among the included patients, and the exploratory nature of the analysis means that further, adequately designed studies are needed to draw definitive conclusions. Moreover, the observed increase in ΔV/Q may primarily reflect relief from over-assistance, and this effect could be more evident in patients with higher PEEP [18]. Indeed, PEEP stabilizes the lung and may accentuate the negative impact of excessive pressure support. If high levels of pressure support and large tidal volumes caused regional overdistension and elevated intrathoracic pressures, reducing pulmonary perfusion, lowering pressure support may have decreased intrathoracic pressures and improved perfusion, with a more evident effect at higher levels of PEEP.

Overall, our study has limitations. First, we acknowledge that the primary outcome measure may provide an overestimate of V/Q matching as the method identifies regions where both ventilation and perfusion are present, without accounting for the proportional relationship between the two (e.g. regions well ventilated but poorly perfused or well perfused but poorly ventilated).

External validity is limited by the small sample size and the descriptive nature of our analyses. The short duration of the observation period may not have allowed detection of all the changes in ventilation and perfusion. Moreover, the study design strongly relies on the P0.1 threshold. The threshold of P0.1 was conservatively adopted considering previous literature [4] and aiming to capture early physiological changes in ventilation and perfusion distribution associated with variations in pressure support. Indeed, recent literature suggests that a P0.1 value above 3.5 cmH₂O may be a sensitive marker of elevated respiratory drive [19] and of potentially injurious transpulmonary pressure swings, while healthy subjects should generate a P0.1 of 1–1.5cmH2O [20]. However, the optimal target range for P0.1 during mechanical ventilation is currently uncertain [20]. Thus, we acknowledge that a different threshold might have led to different results, although the significant separation of other effort-related measures between the two conditions supports the validity of our design. Moreover, clinical improvement and readiness to transition to pressure support ventilation was an inclusion criterion, but it was not protocolized and remained prone to subjectivity of clinical judgement, further limiting the validity of our results.

## Conclusions

Soon after the transition from controlled to assisted mechanical ventilation in patients recovering from ARDS, reducing the level of pressure support determined a redistribution of ventilation that did not, on average, result in improved V/Q matching. Our data underline the need for personalized settings during the transition from controlled to assisted mechanical ventilation in patients recovering from ARDS.

## Supplementary Information

Supplementary material 1.

## Data Availability

The datasets used and/or analysed during the current study are available from the corresponding author on reasonable request.

## Abbreviations

ARDS: Acute respiratory distress syndrome
EIT: Electrical impedance tomography
ICU: Intensive care unit
PEEP: Positive end-expiratory pressure
PMI: Pressure muscle index
PS: Pressure support
Pplat: Plateau pressure
Ppeak_aw_: Peak pressure airway
Pdriving: Driving pressure
Vt: Tidal volume
PBW: Predicted body weight
V/Q: Ventilation/perfusion
