# Supplementary material for: Assessment of V/Q mismatch during pressure support ventilation with electrical impedance tomography: a prospective physiological study
Source: Intensive Care Med Exp. 2025 Dec 11;13:125. doi: 10.1186/s40635-025-00837-6 (PMC12698925; doi:10.1186/s40635-025-00837-6)
Supplement: Supplementary file 1 — Supplementary material 1. [file 40635_2025_837_MOESM1_ESM.docx]

Supplementary Material to:

**Assessment of V/Q mismatch during pressure support ventilation with electrical impedance tomography:**

**A prospective physiological study**

**List of contents**

**Table S1. Radiological findings, primary cause of ARDS and mortality per patient**

**Table S2. Ventilation and perfusion distribution results from EIT data analysis at baseline**

**Table S3. Results of post-hoc sensitivity analysis conducted removing outliers data from the primary outcome analysis**

**Table S4. Ventilation and perfusion distribution results from EIT data analysis at high and low pressure support**

**Figure S1. Graphical visualization of V/Q patient data**

**Figure S2. STROBE Flow Diagram**

**Table S1. Radiological findings, primary cause of ARDS and mortality per patient**

| **ID** | **Radiological findings at ICU admission** | **Cause or suspected cause of ARDS** | **Status at ICU discharge** |
| --- | --- | --- | --- |
| **1** | Chest CT scan: Multiple and diffuse areas of increased parenchymal density with a partly ground-glass and partly consolidative appearance of likely inflammatory nature, more evident in dorsal areas than in ventral areas | Aspiration pneumonia | Alive |
| **2** | Chest CT scan: Multiple and diffuse areas of bilateral lung consolidation | Near drowning syndrome (salted water) | Alive |
| **3** | Chest X-ray: Bilateral clouding of the lung bases, with associated vague areas of consolidation, more pronounced on the left side | Secondary to toxic epidermal necrolysis | Dead |
| **4** | Chest CT scan: Multiple areas of increased parenchymal density, some with centrilobular extension. Small centrilobular abnormalities are also present in the lingula alongside ground-glass areas. Additional ground-glass abnormalities are seen in the left upper lobe. On the right side, there’s a more diffuse distribution of consolidative areas and crazy-paving pattern. | Influenza virus A pneumonia | Alive at ICU discharge but dead at hospital discharge |
| **5** | Chest X-Ray: Diffuse areas of parenchymal consolidation bilaterally | Aspiration pneumonia | Alive |
| **6** | Chest X-Ray: Bilateral opacities in the hilar regions extending to the right lower midfield | Aspiration pneumonia and/or secondary to complicated intrabdominal infection causing septic shock | Dead |
| **7** | Chest X-Ray: Large area of ​​radiopacity with blurred margins in the middle and lower field of the right lobe, slightly present in the remaining lung areas including left side | Legionella pneumonia | Dead |
| **8** | Chest X-Ray: Diffuse and inhomogeneous areas of parenchymal radiopacity with blurred margins and tending towards confluence are found in both hemithoraces, more evident on the left | Legionella pneumonia | Dead |
| **9** | Chest X-Ray: Thickening of the peribronchovascular interstitium of a reticulomicronodular nature with some areas of blurred parenchymal thickening with indistinct margins in both parenchymas | Secondary to complicated intrabdominal infection causing septic shock | Dead |
| **10** | Chest X-Ray: in an almost ubiquitous location, areas of parenchymal consolidation are found, more evident in the left middle lung field, with indistinct margins, tending towards confluence, with the air bronchogram sign in the context | Pneumocystis jirovecii pneumonia | Dead |
| **11** | Chest X-Ray: Faint areas of subopacification with indistinct margins, tending towards confluence, in both lower lung fields bilaterally | Aspiration pneumonia | Alive |
| **12** | Chest CT scan: multiple and ubiquitous areas of increased parenchymal density of mixed appearance, with increased consolidation component and air bronchogram in the context | Influenza virus A pneumonia | Dead |
| **13** | Chest X-Ray: Marked and diffuse pulmonary consolidation bilaterally in all lung fields | Hydrogen sulfide asphyxiation and sewage near drowning syndrome | Alive |
| **14** | Chest X-Ray: bilaterally, the presence of multiple areas of parenchymal consolidation is reported, with air bronchogram in the context, spread in an almost ubiquitous location, tending to confluence and with indistinct margins, more evident in the right middle-upper lung field and in the left middle lung field, where they assume a more consolidative character | Aspiration pneumonia | Alive |
| **15** | Chest X-Ray: Marked and widespread thickening of the peribronchovascular interstitium with a reticular character and an almost ubiquitous distribution associated with some areas of parenchymal consolidation with a predominantly subpleural distribution with partial sparing of the left upper lung field | p-ANCA vasculitis with lung involvement | Dead |

**Table S2. Ventilation and perfusion distribution results from EIT data analysis at baseline**

|  | | Median | | 1st quartile | | 3rd quartile | |
| --- | --- | --- | --- | --- | --- | --- | --- |
| VCV V ventral (non dep) % |  | 63.321 |  | 57.804 |  |  | 65.174 |
| VCV Q ventral (non dep)% |  | 58.855 |  | 49.259 |  |  | 62.674 |
| VCV V dorsal (dep) |  | 36.679 |  | 34.826 |  |  | 42.196 |
| VCV Q dorsal (dep) |  | 41.145 |  | 37.326 |  |  | 50.741 |
| VCV Only V - dead space (%) |  | 24.163 |  | 4.677 |  |  | 28.834 |
| VCV Only Q - shunt (%) |  | 5.263 |  | 4.454 |  |  | 15.000 |
| VCV Only V ventral |  | 13.876 |  | 3.341 |  |  | 23.006 |
| VCV Only Q ventral |  | 1.675 |  | 1.336 |  |  | 4.294 |
| VCV Only V dorsal |  | 5.828 |  | 1.842 |  |  | 10.287 |
| VCV Only Q dorsal |  | 4.032 |  | 3.589 |  |  | 10.526 |
| VCV Matched (%) region |  | 70.574 |  | 58.065 |  |  | 81.579 |
| VCV %V shunt ventral |  | 0.000 |  | 0.000 |  |  | 0.000 |
| VCV %V low ventral |  | 11.041 |  | 10.992 |  |  | 13.941 |
| VCV %V normal ventral |  | 13.945 |  | 11.756 |  |  | 15.182 |
| VCV %V high ventral |  | 20.955 |  | 16.117 |  |  | 26.424 |
| VCV %V dead space ventral |  | 9.831 |  | 1.503 |  |  | 20.418 |
| VCV %V shunt dorsal |  | 0.000 |  | 0.000 |  |  | 0.000 |
| VCV %V low dorsal |  | 8.676 |  | 8.370 |  |  | 11.992 |
| VCV %V normal dorsal |  | 10.313 |  | 7.062 |  |  | 13.891 |
| VCV %V high dorsal |  | 5.656 |  | 3.944 |  |  | 13.281 |
| VCV %V dead space dorsal |  | 3.835 |  | 1.074 |  |  | 7.585 |
| VCV %Q shunt ventral |  | 1.536 |  | 0.535 |  |  | 2.102 |
| VCV %Q low ventral |  | 17.688 |  | 16.281 |  |  | 28.879 |
| VCV %Q normal ventral |  | 13.747 |  | 11.909 |  |  | 15.063 |
| VCV %Q high ventral |  | 14.207 |  | 9.821 |  |  | 14.929 |
| VCV %Q dead space ventral |  | 0.000 |  | 0.000 |  |  | 0.000 |
| VCV %Q shunt dorsal |  | 4.595 |  | 3.127 |  |  | 7.647 |
| VCV %Q low dorsal |  | 17.201 |  | 11.887 |  |  | 19.537 |
| VCV %Q normal dorsal |  | 9.795 |  | 7.173 |  |  | 13.781 |
| VCV %Q high dorsal |  | 3.618 |  | 2.589 |  |  | 7.950 |
| VCV %Q dead space dorsal |  | 0.000 |  | 0.000 |  |  | 0.000 |

Data available for n=5 patients

**Table S3. Results of post-hoc sensitivity analysis conducted removing outliers data (V/Q matched regions < 27%) from the primary outcome analysis**

| **High Pressure Support** | |  | | **Low Pressure Support** | | **p** | **Hodges-Lehmann Estimate** | | |  |
| --- | --- | --- | --- | --- | --- | --- | --- | --- | --- | --- |
| Matched (%) regions  Median (IQR):  50.5 (45.7-69.1) |  |  |  | Matched (%) regions  Median (IQR):  61.8 (60.2-70.5) |  | 0.244 |  |  | -8.173 |  |

*Note: N=13*

**Table S4. Ventilation and perfusion distribution results from EIT data analysis at high and low pressure support**

|  | High Pressure Support | 1st quartile | 3rd quartile | Low Pressure Support | 1st quartile | 3rd quartile | P value |
| --- | --- | --- | --- | --- | --- | --- | --- |
| Only V ventral | 16.402 | 11.033 | 16.402 | 15.056 | 9.668 | 19.688 | 0.599 |
| Only Q ventral | 1.826 | 0.878 | 1.826 | 1.689 | 0.849 | 7.647 | 0.252 |
| Only V dorsal | 11.264 | 5.484 | 11.264 | 14.318 | 7.010 | 20.267 | 0.679 |
| Only Q dorsal | 6.048 | 2.663 | 6.048 | 4.196 | 2.024 | 7.621 | 0.277 |
| %V shunt ventral | 0.000 | 0.000 | 0.000 | 0.000 | 0.000 | 0.000 | NA |
| %V low ventral | 13.828 | 6.698 | 13.828 | 12.429 | 9.138 | 18.855 | 0.804 |
| %V normal ventral | 10.126 | 7.165 | 10.126 | 9.654 | 7.658 | 18.007 | 0.890 |
| %V high ventral | 10.184 | 4.620 | 10.184 | 8.103 | 1.741 | 15.761 | 0.639 |
| %V dead space ventral | 15.329 | 10.938 | 15.329 | 12.589 | 7.818 | 16.332 | 0.524 |
| %V shunt dorsal | 0.000 | 0.000 | 0.000 | 0.000 | 0.000 | 0.000 | NA |
| %V low dorsal | 13.028 | 6.521 | 13.028 | 14.586 | 4.403 | 18.977 | 0.359 |
| %V normal dorsal | 7.707 | 2.969 | 7.707 | 12.671 | 3.149 | 16.401 | 0.421 |
| %V high dorsal | 2.523 | 0.881 | 2.523 | 3.816 | 2.358 | 10.193 | 0.524 |
| %V dead space dorsal | 9.739 | 4.190 | 9.739 | 13.653 | 4.760 | 16.708 | 0.804 |
| %Q shunt ventral | 1.346 | 0.616 | 1.346 | 0.962 | 0.578 | 13.055 | 0.389 |
| %Q low ventral | 23.181 | 10.694 | 23.181 | 24.680 | 15.890 | 32.320 | 0.720 |
| %Q normal ventral | 9.834 | 7.377 | 9.834 | 9.538 | 8.093 | 18.263 | 0.934 |
| %Q high ventral | 6.558 | 3.045 | 6.558 | 5.202 | 1.262 | 9.762 | 0.679 |
| %Q dead space ventral | 0.000 | 0.000 | 0.000 | 0.000 | 0.000 | 0.000 | NA |
| %Q shunt dorsal | 6.249 | 1.522 | 6.249 | 4.418 | 1.633 | 9.641 | 0.720 |
| %Q low dorsal | 22.483 | 13.523 | 22.483 | 22.841 | 9.533 | 32.257 | 0.277 |
| %Q normal dorsal | 8.405 | 3.250 | 8.405 | 12.327 | 3.106 | 17.339 | 0.454 |
| %Q high dorsal | 1.938 | 0.621 | 1.938 | 2.644 | 1.631 | 6.684 | 0.389 |
| %Q dead space dorsal | 0.000 | 0.000 | 0.000 | 0.000 | 0.000 | 0.000 | NA |

**Figure S1. Graphical visualization of V/Q patient data**

**
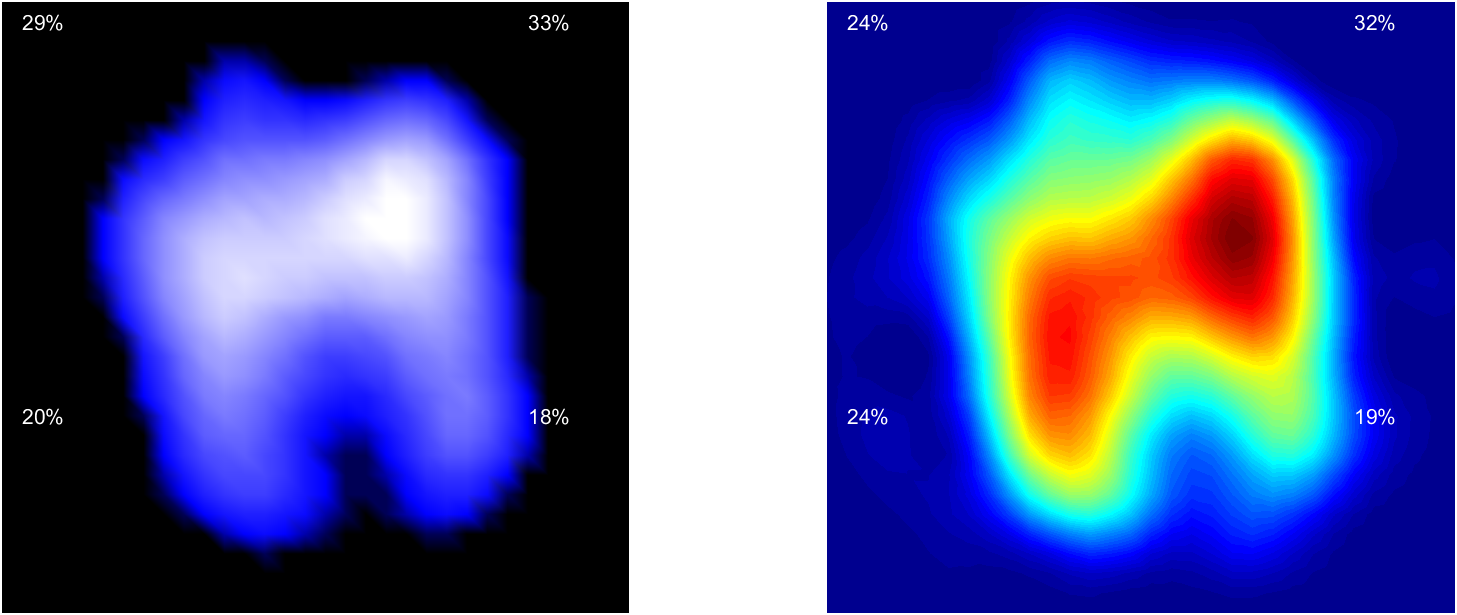
**

The figure shows an example of graphical visualization of a V/Q measurement from EIT patient data. Left panel refers to ventilation and right panel refers to perfusion.

**Figure S2. STROBE Flow Diagram**

**
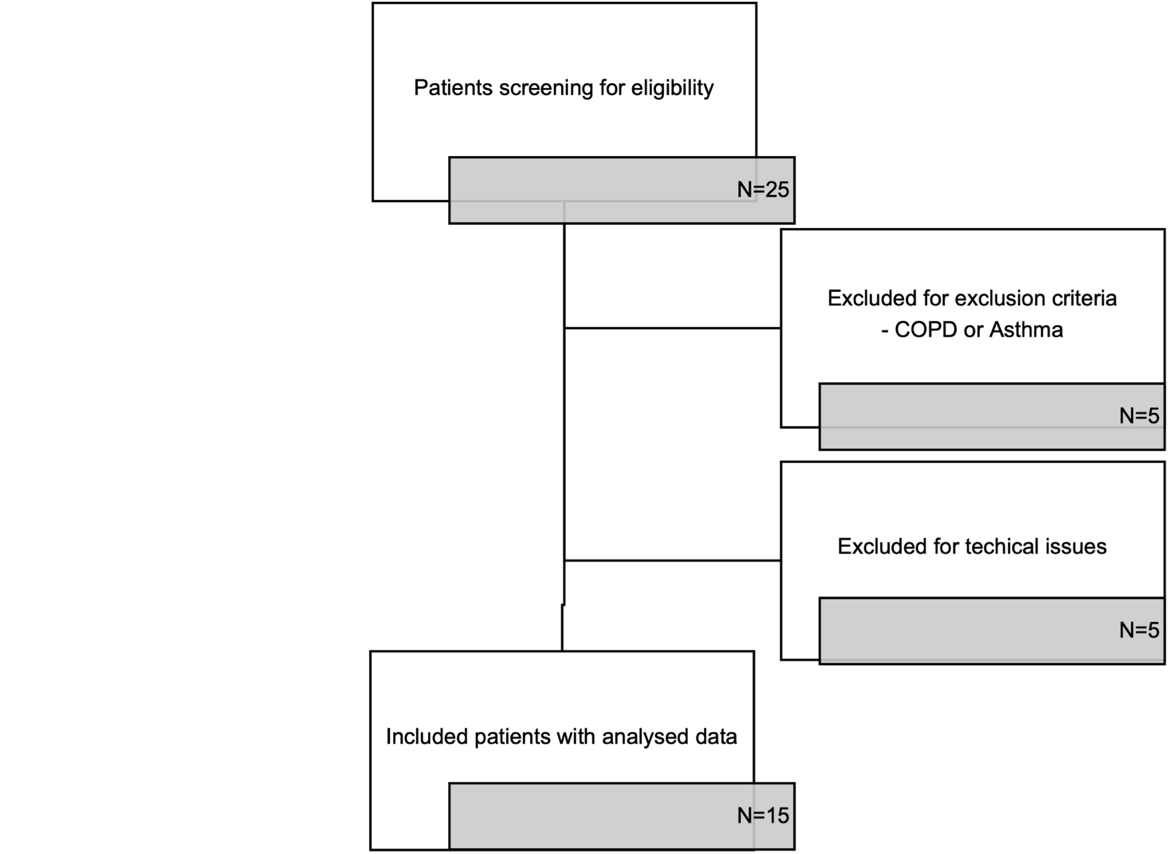
**
